# Efficacy and safety of drug-eluting stenting compared with bypass grafting in diabetic patients with multivessel and/or left main coronary artery disease

**DOI:** 10.1038/s41598-019-43681-x

**Published:** 2019-05-13

**Authors:** Xiaojun Xin, Xiangming Wang, Xuesi Dong, Yuanming Fan, Wei Shao, Xiang Lu, Pingxi Xiao

**Affiliations:** 1 Department of Cardiology, The Affiliated Sir Run Run Hospital of Nanjing Medical University, Nanjing, China; 2 Department of Geriatric Cardiology, First Affiliated Hospital of Nanjing Medical University, Nanjing, China; 3 Department of Biostatistics, School of Public Health, Southeast University, Nanjing, China; 4 Clinical Metabolomics Center, School of Traditional Chinese Pharmacy, China Pharmaceutical University, Nanjing, China

**Keywords:** Interventional cardiology, Cardiac device therapy

## Abstract

Although percutaneous coronary intervention (PCI) with drug-eluting stents (DESs) and bypass grafting are generally believed to be superior revascularization strategies in patients with coronary artery disease (CAD), the optimal strategy for diabetic patients is still controversial. This meta-analysis was performed to compare two methods of revascularization for patients with diabetes mellitus with left main coronary artery lesions or disease in multiple coronary arteries. Compared with the coronary artery bypass grafting (CABG) group, those receiving PCI-DES showed a greater risk of major adverse cardiovascular events (MACEs) (hazard ratio [HR]: 1.12, 95% confidence interval [CI]: 1.01–1.25, *P* = 0.03), major adverse cardiac and cerebrovascular events (MACCEs) (HR: 1.85, 95% CI: 1.58–2.16; *P* < 0.001), stroke (HR: 1.15, 95% CI: 1.02–1.29, *P* = 0.02), myocardial infarction (MI) (HR: 1.48, 95% CI: 1.04–2.09, *P* = 0.03), and repeat revascularization (HR: 3.23, 95% CI: 1.37–7.59, *P* = 0.007). CABG for diabetic patients with multivessel and/or left main CAD was superior to PCI-DES with regard to MACEs, MACCEs, MI, repeat revascularization and stroke, but there was no clear difference in all-cause mortality.

## Introduction

Diabetic patients with coronary atherosclerotic heart disease often have serious and diffuse atherosclerosis of multiple epicardial coronary arteries^1^. Correspondingly, it is necessary for an increasing number of diabetic patients with coronary artery disease (CAD) to undergo drug-eluting stenting with bypass grafting^2^. Diabetes mellitus (DM) is considered a risk factor for a poor prognosis in CAD patients undergoing either percutaneous coronary intervention (PCI) or coronary artery bypass grafting (CABG). In addition, it has been reported that patients with DM could have elevated cardiovascular mortality and morbidity rates after revascularization^3–6^. Although CABG is considered a more appropriate approach to revascularization than PCI in diabetic patients with multivessel coronary disease, it also results in higher rates of adverse cerebral vascular outcomes and perioperative morbidities. Meanwhile, CABG is more invasive than PCI, which causes some patients to choose PCI as a revascularization strategy. Fortunately, with the development of an interventional field, one of the important restrictions on PCI has been substantially reduced. The development of drug-eluting stents (DESs) has clearly decreased the rates of restenosis and repeat revascularizations in comparison with the rates in the previous era of bare metal stents (BMSs)^7–10^.

To date, a number of clinical trials have compared PCI-DES and CABG in patients with DM and multivessel or left main coronary artery disease^11–18^. These trials have elucidated the effects of the two methods on the medium- and long-term clinical endpoints. However, the scale of these observational controlled trials (OCTs) has been small, and some studies were single-centre observational trials. The number of published randomized controlled trials (RCTs) with diabetic patients is currently small.

In summary, there is not powerful evidence to find statistically significant differences with respect to all-cause mortality, major adverse cardiovascular events (MACEs), major adverse cardiac and cerebrovascular events (MACCEs), stroke, repeat revascularization and myocardial infarction (MI) between the PCI and CABG groups. Considering this situation, it is imperative to conduct a quantitative evaluation and synthesis of the current information about the optimal revascularization strategy for these patients.

## Results

### Study selection and characteristics

**Figure 1:**
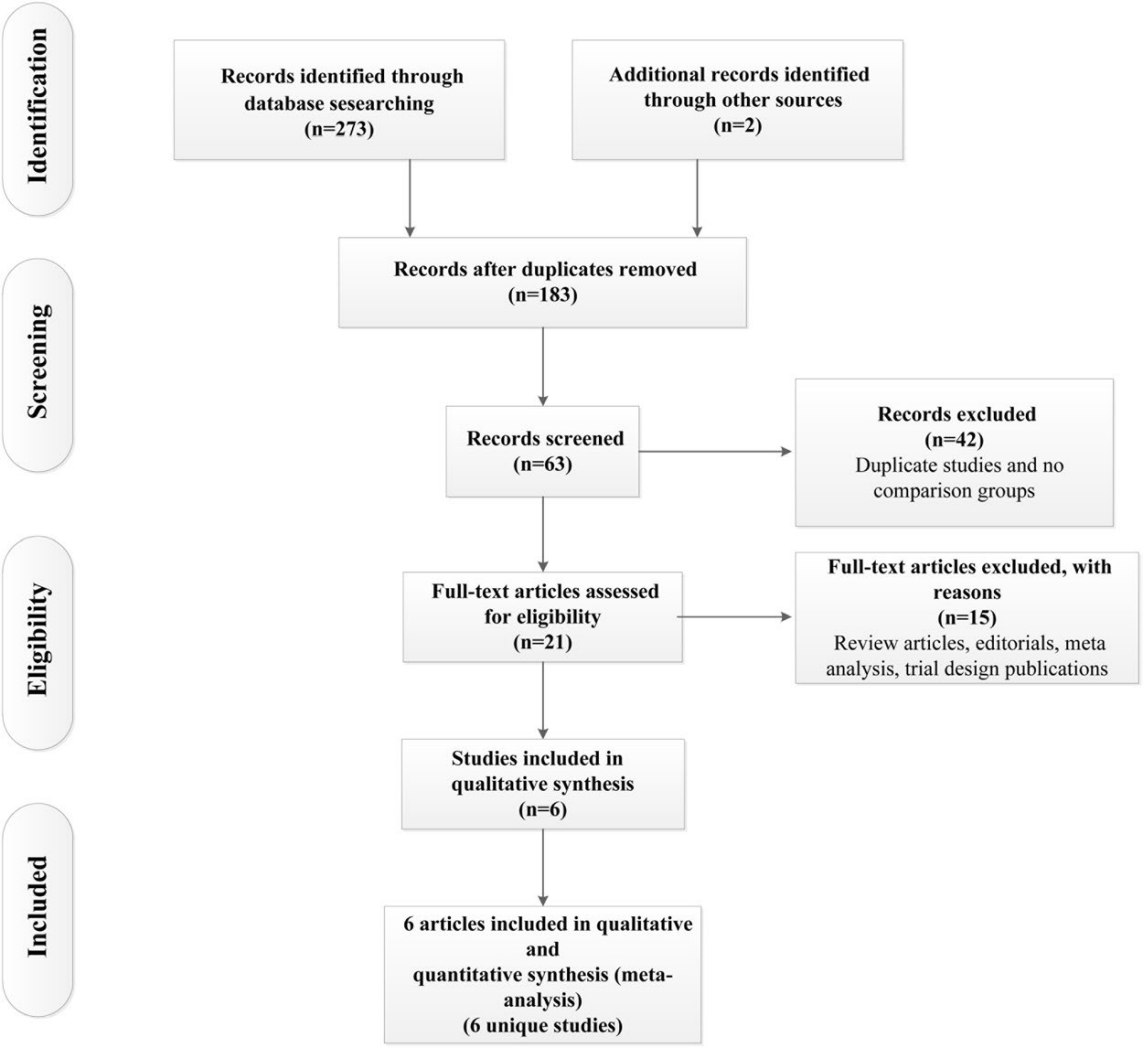
Flowchart for the study selection process.

After removing duplicates and screening at the title and abstract level, 21 potentially qualified studies were identified (Fig. 1). After full-text assessments, we included six clinical trials involving diabetic patients with CAD^11–16^. The 6 eligible articles included 5,013 patients (2,510 patients underwent PCI, and 2,503 patients underwent CABG). The general characteristics of the studies that met the inclusion criteria are summarized in Table 1.

**Table 1:** General characteristics of the included studies.

Study	Location or no. of centre(s)	No. of subjects (PCI versus CABG)	Design	Follow-up duration	Type of revascularization	Coronary lesions
PRECOMBAT^15^	South Korea	102 versus 90	randomized controlled study^#^	5 years	SES/CABG	unprotected left main CAD with stenosis of more than 50%
SYNTAX^11^	85 centres	231 versus 221	randomized controlled study^#^	5 years	PES/CABG	left main CAD (alone or with 1-, 2-, or 3-vessel disease) or isolated 3-vessel disease
FREEDOM^13^	140 centres	953 versus 947	randomized controlled study	5 years	SES or PES/CABG	multivessel CAD with stenosis of more than 70% and without left main coronary stenosis
EXCEL^16^	126 centres	256 versus 249	randomized controlled study^#^	3 years	EES/CABG	left main CAD with stenosis of more than 50%
APPROACH^12^	Canada	869 versus 869	prospective cohort study	12 years	DES*/CABG	multivessel CAD (involving 2 or 3 coronary arteries or left main CAD) and LVD (EF < 50%)
Luo^14^	China	99 versus 127	prospective cohort study	3 years	SES or PES/CABG	left main CAD defined as stenosis ≥ 50% (alone or with 1-, 2-, or 3-vessel disease)

Abbreviations: CAD, coronary artery disease; EF, ejection fraction; LVD, left ventricular dysfunction; SES, sirolimus-eluting stent; EES, everolimus-eluting stent; PES, paclitaxel-eluting stent; CABG, coronary artery bypass grafting; *type of the drug-eluting stents is unknown as drug-eluting stents became available in Alberta on a limited basis in 2003 and were more widely available by 2004; ^#^pre-specified subgroup analysis

### MACE

**Figure 2:**
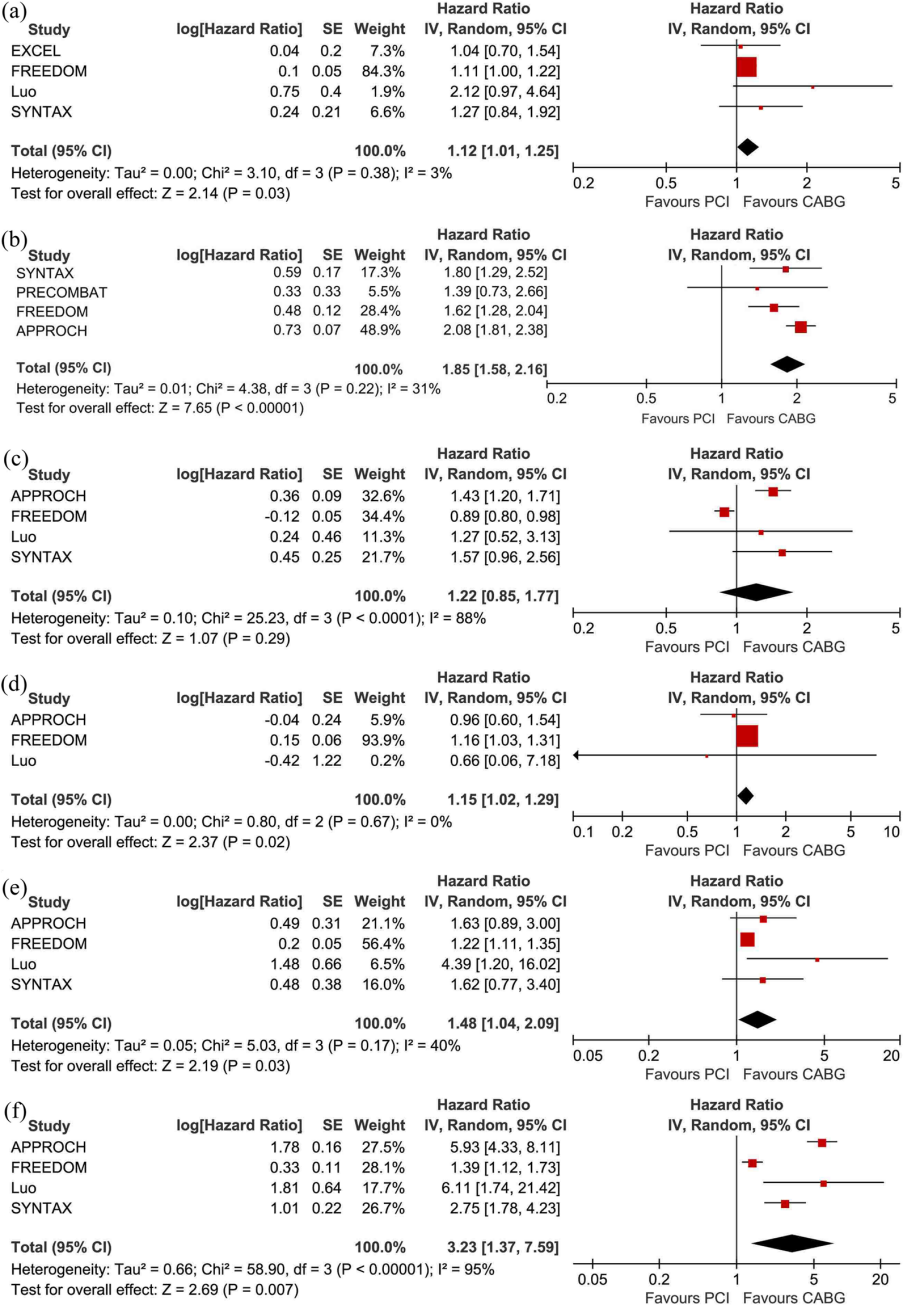
Forest plots for major adverse cardiovascular events (MACE) (**a**), major adverse cardiac and cerebrovascular events (MACCE) (**b**), all-cause mortality (**c**), stroke (**d**), myocardial infarction (MI) (**e**), and repeat revascularization (**f**) in selected trials comparing percutaneous coronary intervention (PCI) versus coronary artery bypass grafting (CABG) in diabetic patients with coronary atherosclerotic heart disease.

**Figure 3:**
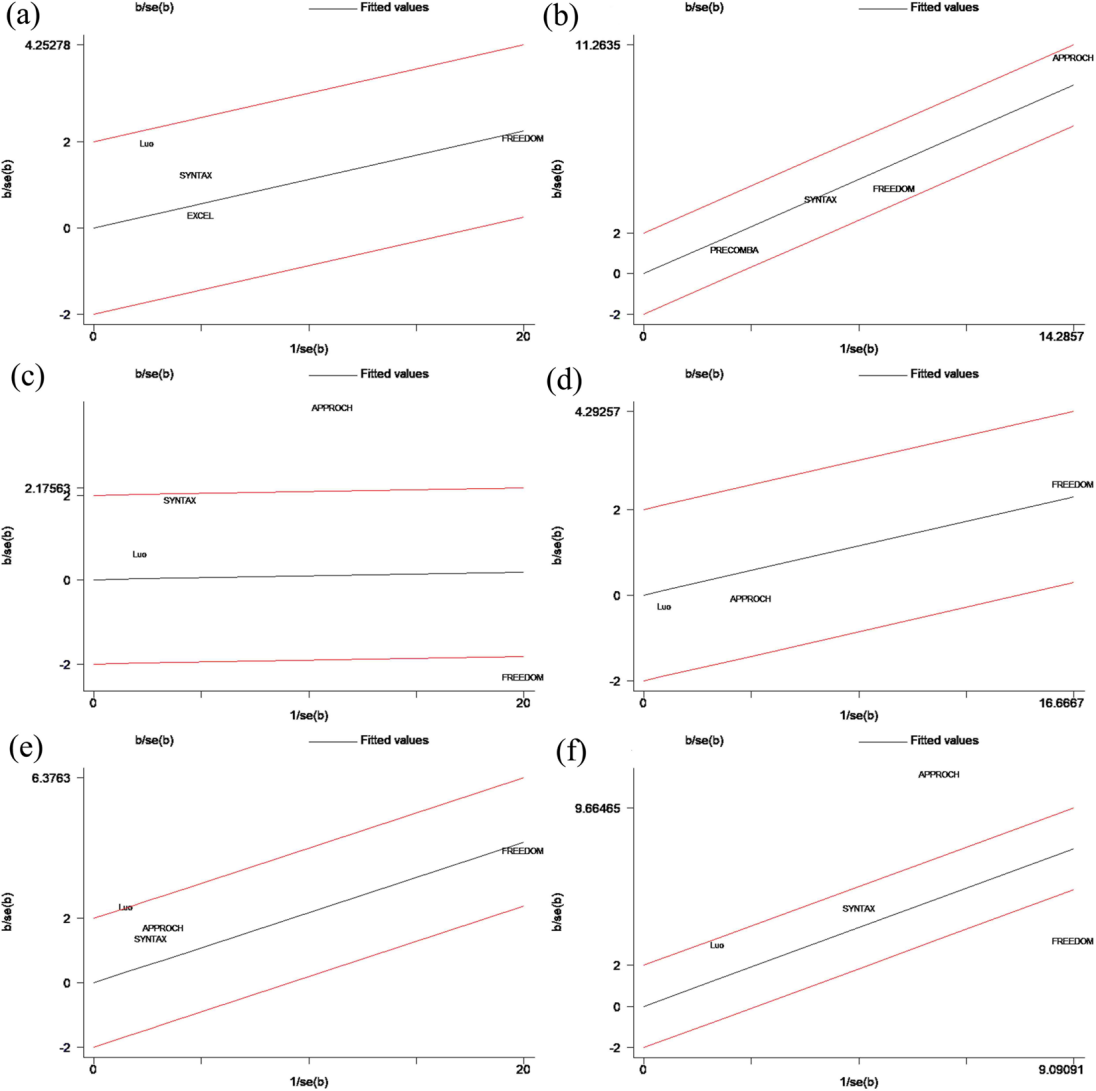
Galbraith plots for major adverse cardiovascular events (MACE) (**a**), major adverse cardiac and cerebrovascular events (MACCE) (**b**), all-cause mortality (**c**), stroke (**d**), myocardial infarction (MI) (**e**), and repeat revascularization (**f**) in selected trials comparing percutaneous coronary intervention (PCI) versus coronary artery bypass grafting (CABG) in diabetic patients with coronary atherosclerotic heart disease.

**Figure 4:**
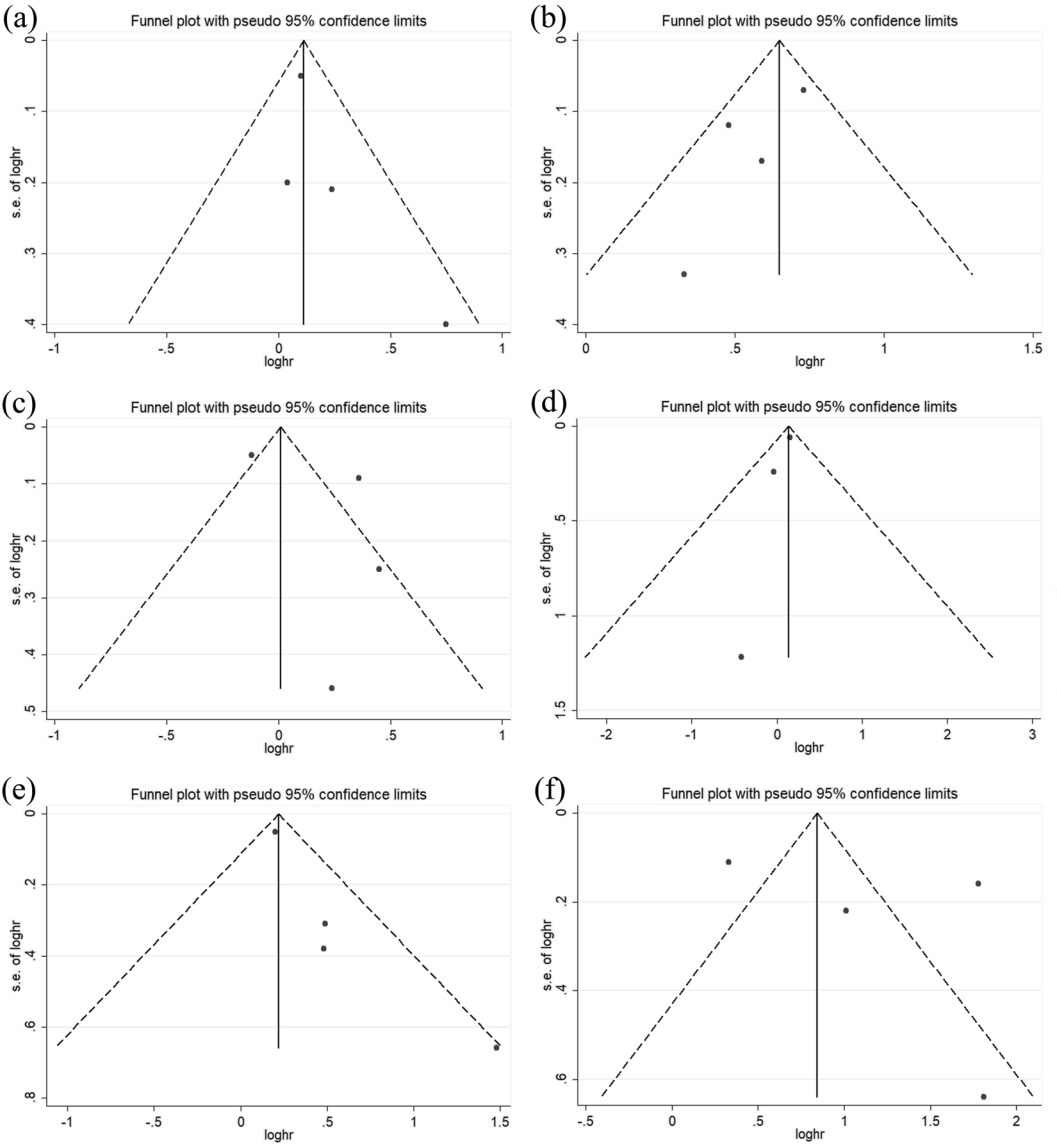
Funnel plots for major adverse cardiovascular events (MACE) (**a**), major adverse cardiac and cerebrovascular events (MACCE) (**b**), all-cause mortality (**c)**, stroke (**d**), myocardial infarction (MI) (**e**), and repeat revascularization (**f**) in selected trials comparing percutaneous coronary intervention (PCI) versus coronary artery bypass grafting (CABG) in diabetic patients with coronary atherosclerotic heart disease.

**Figure 5:**
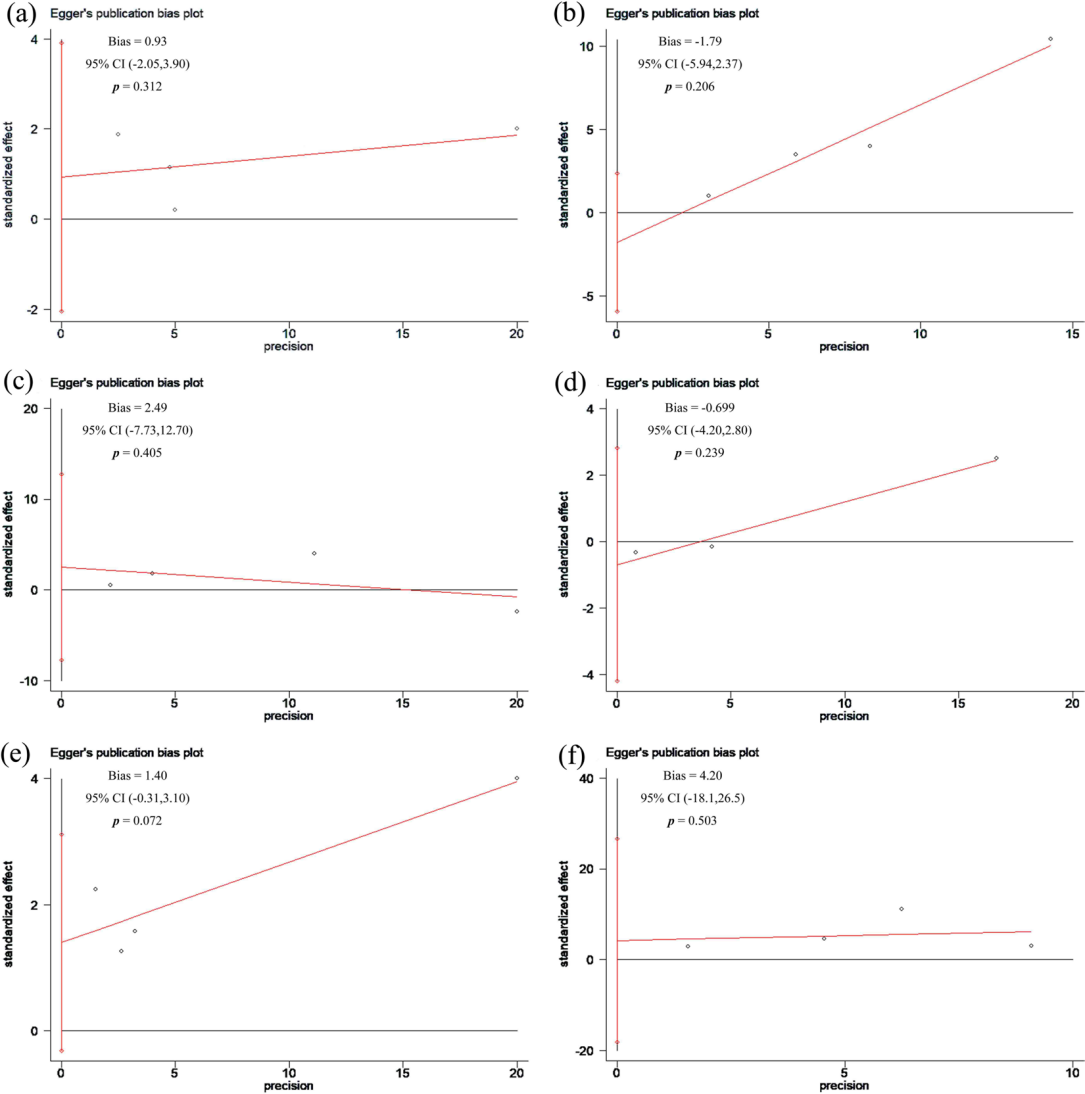
Egger’s publication bias plots for major adverse cardiovascular events (MACE) (**a**), major adverse cardiac and cerebrovascular events (MACCE) (**b**), all-cause mortality (**c**), stroke (**d**), myocardial infarction (MI) (**e**), and repeat revascularization (**f**) in selected trials comparing percutaneous coronary intervention (PCI) versus coronary artery bypass grafting (CABG) in diabetic patients with coronary atherosclerotic heart disease.

Compared with patients undergoing CABG, those undergoing PCI-DES experienced more MACEs (HR: 1.12, 95% CI: 1.01–1.25, *P* = 0.03). There was low heterogeneity among the studies with respect to the composite endpoints (I^2^ = 3%, *P* = 0.38) (Figs 2(a) and 3(a)). There was no obvious publication bias with regard to MACEs revealed by either the visual assessment of the funnel plot or the quantitative evaluation with Egger’s test (*P* = 0.31) (Figs 4(a) and 5(a)). The sensitivity analysis via a random effects model indicated that the superiority of CABG over PCI was clear, with no statistically significant difference compared with the results of the fixed effects meta-analysis (Table 2).

**Table 2:** Sensitivity analysis.

	Outcome (total included patients)	HR (95% CI)	*P* value	Heterogeneity
Analysis performed via a fixed effects model	MACE (N = 3083)	1.12 (1.02, 1.23)	*P* = 0.02	I² = 3%, P = 0.38
	Stroke (N = 3864)	1.15 (1.02, 1.29)	*P* = 0.02	I² = 0%, P = 0.67
Analysis after excluding APPROACH trial performed via a random effects model	MACCE (N = 2544)	1.65 (1.37, 1.99)	*P* < 0.001	I² = 0%, P = 0.75
Analysis after excluding FREEDOM trial performed via a random effects model	All-cause mortality (N = 2416)	1.44 (1.22, 1.70)	*P* < 0.001	I² = 0%, P = 0.91
Analysis after excluding Luo trial performed via a random effects model	MI (N = 4090)	1.24 (1.12, 1.36)	*P* < 0.001	I² = 0%, P = 0.51
Analysis after excluding APPROCH and FREEDOM trials performed via a random effects model	Repeat revascularization (N = 678)	3.27 (1.71, 6.24)	*P* = 0.003	I² = 28%, P = 0.24

Abbreviations: HR, hazard ratio; CI, confidence interval; MACE, major adverse cardiovascular events; MACCE, major adverse cardiac and cerebrovascular events.

### MACCE

MACCEs were reported in 4 of the 6 included articles. Compared with patients undergoing PCI-DES, those undergoing CABG had significantly fewer MACCEs (HR: 1.85, 95% CI: 1.58–2.16; *P* < 0.001). There was moderate heterogeneity (I^2^ = 31%, *P* = 0.22) among the trials with respect to this outcome as revealed by the forest plot (Fig. 2(b)). This moderate heterogeneity in the trials was largely due to the inclusion of the APPROACH trial, as seen in the Galbraith plot (Fig. 3(b)). After removing the APPROACH trial, an HR of 1.65 (95% CI 1.37–1.99) was obtained through a random effects model for this end point (*P* < 0.001) without residual heterogeneity (I^2^ = 0%, *P* = 0.75) (Table 2). Visual evaluation of the funnel plot and quantitative estimation with Egger’s test (*P* = 0.21) indicated no significant publication bias with regard to this endpoint (Figs 4(b) and 5(b)).

### All-cause mortality

There was no statistical evidence of a difference in all-cause mortality (HR: 1.22, 95% CI: 0.85–1.77, *P* = 0.29) (Fig. 2(c)). Through the heterogeneity analysis, a high degree of heterogeneity was found in the included trials (I² = 88%, *P* < 0.001) due to the inclusion of the FREEDOM trial or the APPROACH trial according to visual inspection of the Galbraith plot (Fig. 3(c)). When the FREEDOM trial was excluded, the random effects model for all-cause mortality had an HR of 1.44 (95% CI: 1.22–1.70, *P* < 0.001, I^2^ = 0%, *P* = 0.91) (Table 2). Studies of all sizes unequally distributed on both sides of the line and expanded past the 95% confidence limits in the funnel plot (Fig. 4(c)), although no publication bias was indicated by the quantitative estimation with Egger’s test (*P* = 0.41) (Fig. 5(c)). This indicated that there were other reasons causing the asymmetry of the funnel plot.

### Stroke

Compared with patients undergoing CABG, those undergoing PCI-DES had a higher risk of stroke (HR: 1.15, 95% CI: 1.02–1.29, *P* = 0.02, I^2^ = 0%, *P* = 0.67) (Fig. 2(d)). There was no publication bias according to the results of Egger’s test (*P* = 0.24) (Fig. 5(d)) and the visual inspection of the funnel plot (Fig. 4(d)). The sensitivity analysis with a fixed effect model also suggested that the incidence of stroke was higher in the PCI-DES group than in the CABG group (HR: 1.15, 95% CI: 1.02–1.29, *P* = 0.02) (Table 2).

### Myocardial infarction

Compared to the CABG group, the PCI group had a higher rate of MI (HR: 1.48, 95% CI: 1.04–2.09, *P* = 0.03). The moderate heterogeneity (I^2^ = 40%, *P* = 0.03) with respect to this endpoint among the trials is shown in Fig. 2(e). The heterogeneity was mainly due to the inclusion of the Luo study, according to the Galbraith plot (Fig. 3(e)). Therefore, we performed a sensitivity analysis with a random effects model by excluding the Luo trial. The result (HR: 1.24, 95% CI: 1.12–1.36, *P* < 0.001, I² = 0%, *P* = 0.51) (Table 2) was significantly different from the result of the previous analysis, which included the Luo trial. There was no significant publication bias according to the results of Egger’s test (*P* = 0.07) (Fig. 5(e)) and the funnel plot (Fig. 4(e)).

### Repeat revascularization

Compared with patients undergoing CABG, those undergoing PCI had a higher risk of repeat revascularization (HR: 3.23, 95% CI: 1.37–7.59, *P* = 0.007). There was a high degree of heterogeneity (I^2^ = 95%, *P* = 0.007) among the trials with respect to this outcome (Fig. 2(f)), which was mainly due to the APPROACH trial and FREEDOM trial according to visual inspection of the Galbraith plot (Fig. 3(f)). After the exclusion of these two trials, a sensitivity analysis with a random effects model yielded an HR of 3.27 (95% CI: 1.71–6.24, *P* = 0.003) for repeat revascularization with moderate heterogeneity (I² = 28%, *P* = 0.24) (Table 2). Egger’s test (*P* = 0.50) (Fig. 5(f)) and inspection of the funnel plot (Fig. 4(f)) indicated that there was no clear publication bias among the trials.

## Discussion

In this meta-analysis incorporating 5,013 diabetic patients with multivessel and/or left main CAD, compared with patients undergoing PCI-DES, those undergoing CABG had lower incidence rates of MACEs, MACCEs, MIs, strokes and repeat revascularization. However, there was no clear difference between the two groups with regard to all-cause mortality. The observed superiority of CABG to PCI-DES with regard to MACEs was consistent in all included studies, with little heterogeneity. Meanwhile, we found that compared with patients undergoing CABG, those undergoing PCI had a significantly higher prevalence of MACCEs, with moderate heterogeneity mainly driven by the APPROACH trial. There was no publication bias regarding any clinical endpoint according to Egger’s test.

Furthermore, our study was dominated by two large trials (APPROACH trial and FREEDOM trial), which accounted for 73% of the included patients in this analysis. The first data provided by the APPROACH trial showed that the diabetic population with CAD and left ventricular dysfunction (LVD) benefit from CABG rather than PCI-DES, with an elevated long-term overall survival and reduced prevalence rates of repeat revascularization and MI; no clear difference was found with respect to stroke among the included diabetic patients. Several previous RCTs mainly recruited diabetic patients without LVD or included patients who were nonrepresentative. The APPROACH trial fills this gap in the literature^12,13,19,20^. The FREEDOM trial is the largest randomized trial comparing PCI and CABG in a population with DM and multivessel disease (MVD). In this trial, we observed that CABG was superior to PCI-DES in diabetic patients; compared with patients undergoing CABG, those undergoing PCI had significantly higher rates of mortality and MI, although they had a decreased incidence of stroke. Meanwhile, we did not detect a significant difference with respect to all-cause mortality in the FREEDOM trial^13^. However, the controversy about the two strategies continued. A subgroup analysis of the SYNTAX trial for patients with DM, LM and/or MVD indicated that an elevated risk of repeat revascularization was present in the diabetic subgroup in the PCI arm (HR: 2.75, 95% CI: 1.78–4.24, *P* < 0.001), and a substantially elevated rate of MI was observed in the PCI subgroup of diabetic patients (HR: 1.62, 95% CI: 0.77–3.41, *P* = 0.20). However, there was no significant difference in MACEs or all-cause mortality in the diabetic subgroup.

It is noteworthy that the clinical endpoints after PCI were substantially worse for patients with DM than for patients without DM. In the DM population, atherosclerotic lesions may occur in two or three coronary arteries. Additionally, the typical diffuse atherosclerosis with stenosis in multiple locations could result in a reduced success rate of PCI and an elevated risk of restenosis in diabetic patients^21^. Meanwhile, multiple stenoses means that two or more stents need to be placed in the coronary arteries. This increases the difficulty of PCI and complicates the surgery. To date, the precise mechanism underlying the inferiority of PCI to CABG in diabetic patients is not clear. However, the greater chance of restenosis after PCI, which leads to repeat revascularization and more aggressive progression of atherosclerosis in segments in which stents are not placed, may in part explain these different revascularization endpoints in diabetic patients^22–25^.

The presentation of diabetic patients appeared to strengthen the superiority of CABG over PCI. CABG is considered a more appropriate approach to revascularization in patients with DM, LM and MVD. However, in clinical practice, PCI is the preferred initial revascularization strategy for diabetic patients because compared with CABG, it is less invasive, requires a shorter time for recovery and carries lower rates of wound infection, bleeding and arrhythmia. Meanwhile, a clear disadvantage of CABG compared with PCI is the occurrence of complications such as repeat thoracotomy for bleeding, postoperative pneumonia, permanent pacemaker implantation, pleural effusions or ventricular fibrillation, which affect 50% of diabetic patients after CABG^26^.

Ultimately, the aim of a meta-analysis that combines the results of prior studies is to draw a clear conclusion when previous individual trials were unable to generate convincing results. This study summarized the safety and effectiveness of the two methods of revascularization in diabetic patients. In addition, we compared the two revascularization strategies in observational controlled studies that included real-world patients and RCTs that included selected patients. Meanwhile, in this meta-analysis, it was appropriate to use HRs for the time-to-event outcomes, which considered the number and timing of events, and the time until the last follow-up for each patient who has not experienced an event and has therefore been censored^27^. In most previous published meta-analysis, odds ratios (ORs) or relative risks (RRs), which are suitable for analysing dichotomous outcomes, were adopted more frequently, although they are less appropriate than HRs for estimating time-to-event results^28^.

### Limitations of the study

We acknowledge several limitations of this meta-analysis. First, there were some inherent biases and limitations, such as publication bias, that existed in two of the OCTs included in our meta-analysis^29,30^. Considerable heterogeneity among the included trials with respect to both the design characteristics and the effect sizes of each clinical outcome were found in this article. Second, in-depth risk stratification on the basis of patients’ complicacy of anatomy was not performed, although the appropriate revascularization strategy changed according to coronary complicacy, as indicated in the SYNTAX trial. Finally, we did not perform an analysis of clinical short-term outcomes in our study because it was difficult to comprehensively estimate the safety and effectiveness of the two revascularization methods in the short-term.

### Conclusions

PCI-DES was inferior to CABG for diabetic patients with left main CAD and/or MVD with regard to MACEs, MACCEs, MIs, strokes and repeat revascularization. However, no obvious difference was observed in all-cause mortality.

## Methods

### Search strategy

We conducted a literature search in PubMed, EMBASE, and the Cochrane Central Register of Controlled Trials for relevant clinical studies from January 2003 to May 2018 without language or publication limitations. Both searches of the title/abstract and Medical Subject Heading terms, including ‘left main’, ‘multivessel disease’, ‘coronary artery bypass’, ‘percutaneous coronary intervention’, ‘drug-eluting stents’, and ‘diabetes mellitus’, were used to optimize the search strategy and identify all relevant clinical trials comparing interventional therapy with bypass operation on coronary arteries in a diabetic subgroup. Meanwhile, we identified additional studies through the references of review articles.

### End points

The primary end point was the occurrence of MACCEs, which were defined as death from any cause, MI, stroke, or repeat revascularization. MACEs comprised death from any cause, MI, and stroke. The secondary end points included each MACE component.

### Selection criteria

We selected the eligible studies that met the following criteria: 1) clinical trials that included patients with DM and LM and/or MVD; 2) comparison of PCI-DES versus CABG; 3) RCTs, OCTs and accessible articles with pre-established subgroup analyses; and 4) clinical follow-up times equal to or greater than twelve months. Studies were considered ineligible according to the following criteria: 1) DESs were not used during interventional therapy; 2) the number of patients included in each arm was less than 50; and 3) repetitive studies.

### Study selection

After removing the duplicate studies, the citations and titles/abstracts of the publications identified by the search strategy were screened independently by two reviewers. The full texts of the studies considered relevant by the two reviewers were then retrieved and reviewed. Disagreements with regard to the inclusion of studies during the full-text review were settled by discussion between the two reviewers.

### Data extraction

Available data were extracted from the selected trials by two researchers who worked independently. Disagreements were determined via consensus or by consultation with a third researcher when necessary. The following information was extracted from the eligible studies: the name of the study, location or number of centres, number of patients randomized in each arm, duration of the clinical follow-up, design of research, type of revascularization, and characteristics of the coronary lesions. The following primary clinical outcome events were also extracted: MACEs, MACCEs, MIs, all-cause mortality, repeat revascularization, and stroke. Data were extracted from complete articles, and other relevant literature was used as supplementary materials when the articles were reported in >1 publication. If the relevant outcomes were not provided in the eligible articles, we also attempted to contact the corresponding author by e-mail to obtain the details.

### Statistical methods

We meta-analysed all endpoints of the selected articles using HRs with 95% CIs. The overall effect size was estimated via Der Simonian and Laird random effects models^31^. To estimate the heterogeneity, the Cochran Q test with a Mantel – Haenszel test on the basis of the pooled HRs was adopted across the studies. The I^2^ statistic was used to assess the between-trial heterogeneity (ensuring the variance across arms was a result of heterogeneity rather than chance)^32^. Values of 25%, 50%, and 75% were regarded as low, moderate, and high degrees of heterogeneity on the basis of the I^2^ statistic^33^. Galbraith plots were also used as a measure to evaluate the heterogeneity of the publications by graphical means. Meanwhile, we constructed forest plots to graphically show the endpoints in clinical practice. Sensitivity analyses were conducted via fixed effects or random effects models with inverse-variance weighting in our repeated analysis. Funnel plots and Egger’s tests (*P* > 0.05) were used to visually estimate the publication bias. HRs were converted to a logarithmic scale for the funnel plot. In the absence of publication bias, trials are dispersed equally on both sides of the line that evaluates the natural log of the HR. When *P* is > 0.05 for Egger’s publication bias plot, the included trials were considered to have no publication bias. We used STATA 14 which was purchased by Nanjing medical university school of public health or Reviewer Manager software (Version 5.3. Copenhagen: The Nordic Cochrane Centre, The Cochrane Collaboration, 2014), where appropriate, to perform the statistical analyses.
